# Brain Dead and Pregnant

**DOI:** 10.7759/cureus.44172

**Published:** 2023-08-26

**Authors:** Natalia Moguillansky, Michael Mathelier, Ibrahim S Tuna

**Affiliations:** 1 Department of Medicine, Division of Pulmonary Critical Care and Sleep Medicine, University of Florida Health, Gainesville, USA; 2 Department of Emergency Medicine, University of Florida College of Medicine, Gainesville, USA; 3 Department of Radiology, University of Florida College of Medicine, Gainesville, USA

**Keywords:** panhypopituitarism, gestational diabetes insipidus, intracranial hemorrhage (ich), brain death critical care, high-risk pregnancy, brain death diagnosis

## Abstract

The presence of pregnancy in a brain-dead woman is a rare circumstance. We present a case of a 31-year-old woman who was 22 weeks pregnant at the time of diagnosis of brain death after intracranial and subarachnoid hemorrhage. After a multidisciplinary approach, the decision was made to continue somatic support to maintain the pregnancy until optimal fetus viability. Cesarean section was performed after 11 weeks (33 weeks gestational age) of brain-death diagnosis with a successful delivery of a live infant. Management of brain-death complications during pregnancy is described.

## Introduction

The combination of pregnancy in a brain-dead woman is an exceptional circumstance. A recent systematic review of the literature on brain-dead patients during pregnancy by Dodaro et al. identified only 35 similar cases [1]. The complexity of brain death in pregnancy requires a multidisciplinary approach and somatic support of the brain-dead mother carries significant complications. These complications include, but are not limited to infections, cardiac arrhythmias, diabetes insipidus, panhypopituitarism, disseminated intravascular coagulation, hypotension, neurogenic pulmonary edema, and systolic myocardial dysfunction [2]. We present a case of a 31-year-old woman who was 22 weeks pregnant at the time of diagnosis of brain death after intracranial and subarachnoid hemorrhage. Management of brain death complications and its ethical and legal aspects are described and reviewed.

## Case presentation

**Figure 1:**
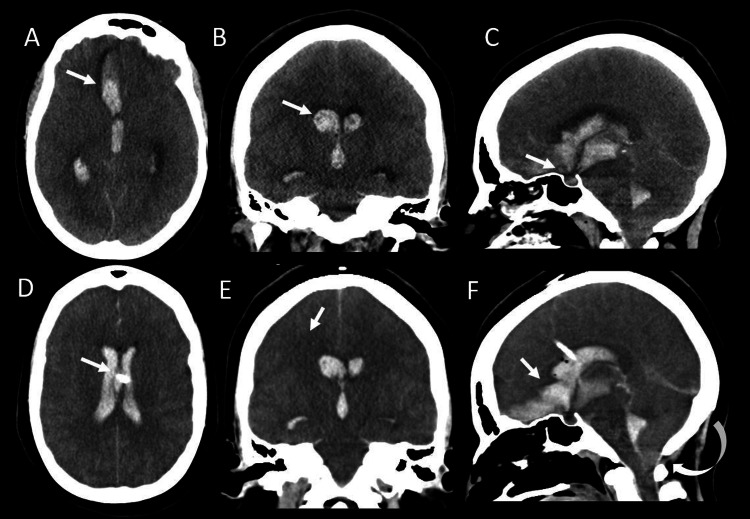
Nonenhanced computed tomography of the head Axial (A) nonenhanced CT with coronal (B) and sagittal (C) reformats demonstrates right inferior frontal lobe parenchymal hemorrhage (arrow in A) with large right more than left intraventricular hemorrhage (arrow in B) and mild subarachnoid hemorrhage (arrow in C). Right more than left ventriculomegaly. Follow-up CT head (D, E, F) without contrast demonstrates interval placement of a ventriculostomy catheter (arrow in D) and persistent diffuse narrowing of cerebral sulci with diffuse brain swelling. Early decreased gray-white matter differentiation, which is more conspicuous in the basal ganglia (arrow in E). Demonstrated right frontal parenchymal hemorrhage with large intraventricular and subarachnoid hemorrhage, which has enlarged intervals (arrow in F). There is early transtentorial and tonsillar herniation (curved arrow in F).

A 31-year-old woman, gravida 13, para 6, with a past medical history of hypertension who was 22 weeks pregnant presented to an outside facility with a severe, acute-onset headache. At the outside facility, she had seizure-like activity, became unresponsive with a Glasgow Coma Score (GCA) of 3 (E1V1M1), and was emergently intubated without the use of sedative or paralytic drugs. Computed tomography (CT) head showed right intraparenchymal hemorrhage with intraventricular and subarachnoid hemorrhage. Possible impending herniation and ventriculomegaly (Figure 1) in a pattern suggesting anterior communicating artery rupture. She was transferred to our facility five hours after the onset of symptoms.

On presentation to our emergency department, her oxygen saturation was 95% on the ventilator. Ventilator settings were synchronized intermittent mandatory ventilation (SIMV) with a respiratory rate of 12, positive end-expiratory pressure (PEEP) of 5, tidal volume of 400, and a fraction of inspired oxygen (FiO2) of 40% (her ventilator and oxygen requirements remained stable throughout her hospitalization). Her blood pressure was 139/97 mmHg, and her temperature was 36.6 with a pulse of 86 beats per minute. Her lungs and cardiac examination were unremarkable. She was unresponsive while off sedation, pupils were 4 mm and non-reactive. She had no gag or cough reflexes that remained unchanged throughout the hospitalization (E1V1M1). An external ventricular drain was placed, and she was admitted to the neurointensive care unit.

**Figure 2:**
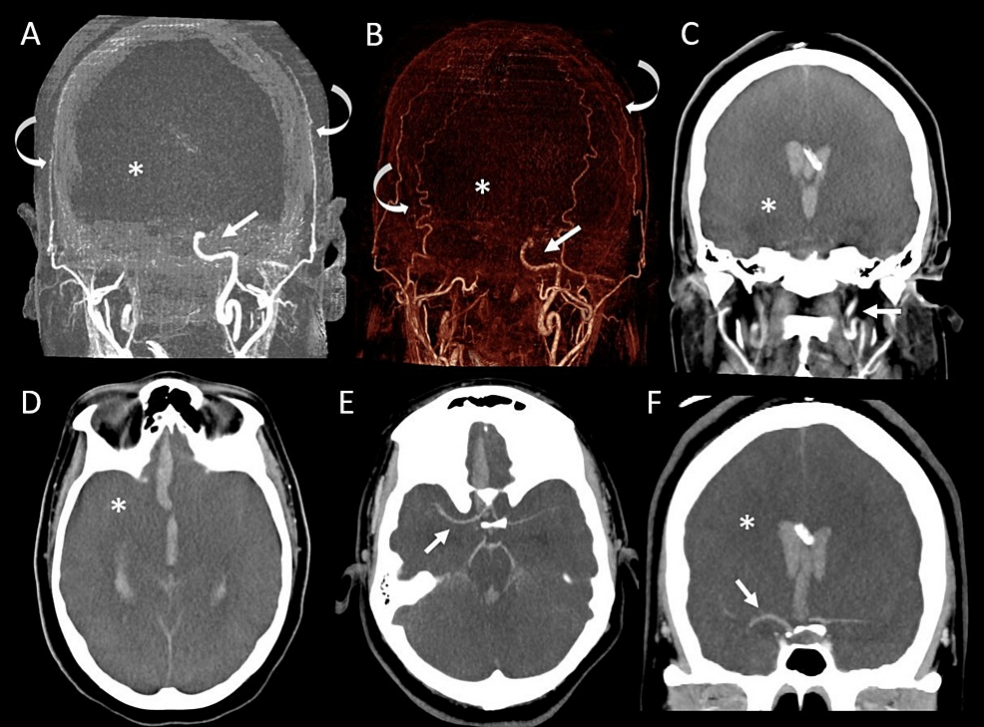
Arterial phase head computed tomography (CTA) Arterial phase CTA head in coronal subtracted (A) maximum intensity projection (MIP) and coronal volume rendered image (B) and coronal (C) and axial (D) MIP demonstrate lack of opacification of the intracranial arteries (asterisk in A, B, C, D) beyond the left cavernous internal carotid artery; although there is contrast opacification of the external carotid artery branches (curved arrows in A, B) likely due to high intracranial pressure. Delayed postcontrast CTA in axial (E) and coronal (F) MIP demonstrates only minimal filling of the proximal intracranial arteries (arrows in E, F), which are diffusely narrowed with a lack of contrast opacification in the distal intracranial arteries (asterisk in F) representing diffuse cerebral swelling and high intracranial pressures suggesting early brain death.

Follow-up computed tomography angiography (CTA) head and neck with/without intravenous contrast showed slightly increasing large right frontal intraparenchymal hemorrhage, which ruptured to the ventricle with subarachnoid hemorrhage and intraventricular hemorrhage throughout the ventricular system (Figure 2). There was diffuse cerebral edema and severely narrowed visualized most proximal intracranial arteries and minimal blood flow seen on delayed contrast imaging, with a lack of blood flow to distal intracranial arteries on the arterial and delayed angiographic phase (Figure 2). There was also likely transtentorial and tonsilar herniation. A ruptured aneurysm was not confirmed on CTA; however, evaluation was limited due to a lack of expected contrast opacification of intracranial arteries due to brain swelling and increased intracranial pressure. These findings were compatible with severe diffuse hypoxic-ischemic injury and concerning for early brain death.

**Figure 3:**
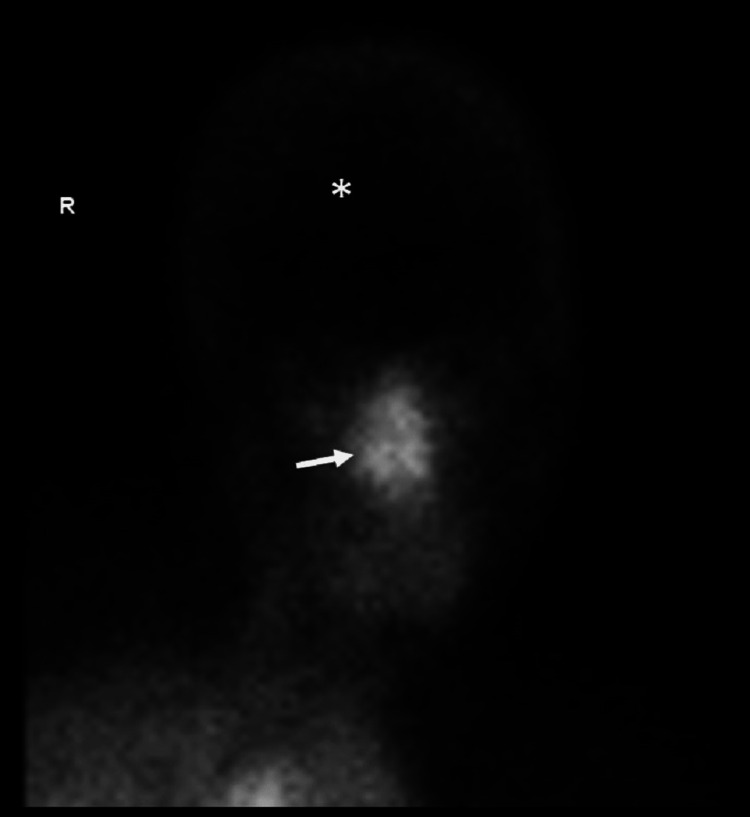
Nuclear medicine brain scan Follow-up Tc-99m HMPAO scan shows absent cerebral perfusion (asterisk), which met the nuclear medicine criteria for brain death. Relatively increased nasal region perfusion representing a hot nose sign (arrow) due to preserved external carotid artery flow but absent brain perfusion and visualization on sagittal sinus.

Due to the findings on the above CT head and neck, a protocol for brain death evaluation was done. She had normal vital signs, sodium was 144 mmol/L, and the urinary drug screen was negative. An apnea test was not performed due to a viable fetus. A nuclear medicine brain scan was done, and it showed intact perfusion up the common carotid arteries, but no intra-cerebral perfusion was identified. The sagittal sinus was not visualized. Next, an immediate three-minute "static" picture of the brain was obtained, showing similar findings with no sagittal sinus visualization. Scan findings met the nuclear medicine criteria for “brain death” and the patient was declared dead on the day of admission (Figure 3).

Her pregnancy appeared normal on a viability scan performed on presentation with a fetal heart rate of 140 beats per minute (bpm) and normal fetal movements at 22 weeks of gestational age. Betamethasone for fetal lung maturity was given on days 9 and 10 of admission. After a multidisciplinary meeting with neurointensivists, neonatal ICU specialists, obstetricians, as well as legal, ethics, and social worker teams, the patient’s family expressed the desire to maintain the pregnancy and bring the fetus to viability by continuing somatic support of the patient.

On the day of admission, she became hypotensive. For this reason, norepinephrine and vasopressin drips were started. Also, on the day of admission, a nasogastric feeding tube was placed. On day 3 of admission, a percutaneous tracheostomy was performed. On day 4, a peripherally inserted central catheter (PICC line) was placed. A radial arterial line was also introduced.

Tube feedings were started after admission (vital 1.5) at 45 ml/hour with two packets of ProSource and free water flushes of 10 ml/hour (free water was titrated for sodium and urine output). Tube feedings continued throughout hospitalization until the patient had a cesarean section.

Eight days after admission, she was transferred to the medical intensive care unit (MICU) for further management with fetal monitoring by the obstetrics service. The obstetric nurse was at the bedside continuously monitoring the fetal heart rate and movements. She remained in the MICU for 11 weeks until her cesarean section on week 33 of gestation age.

On day 3 of admission, endocrinology was consulted for the management of adrenal insufficiency, diabetes insipidus, and central hypothyroidism. For adrenal insufficiency, she was started on stress dose steroids with hydrocortisone 50 mg IV every six hours due to low adrenocorticotropic hormone (ACTH) (<5 pg/ml) and AM cortisol (2.7 mcg/dl). For the treatment of diabetes insipidus, she was treated with vasopressin. Sodium level was 145 mmol/L on day 3 and a range of 135-156 mmol/L throughout hospitalization. Urine output was between 100-200 ml/hour and maintained by titrating vasopressin and free water flushes. For the evaluation of central hypothyroidism, she had her thyroid stimulating hormone (TSH; range 0.4-5 mIU/L) and free T4 (range 0.6-1.2 ng/dL) levels checked. TSH levels were 5.5 mIU/L on admission, 0.61 mIU/L on day 3, and 0.09 mIU/L on day 10. Free T4 on admission was 0.83 ng/dL and 0.76 ng/dL on day 3. She was started on IV levothyroxine 50mcg daily on day 7. Her free T4 was 0.54 ng/dl on day 10 so IV levothyroxine was increased to 62.5 mcg daily. Free T4 remained above 0.6 ng/dL throughout hospitalization after day 14. She also had steroid-induced hyperglycemia for which she was treated with subcutaneous insulin.

On day 4 of admission she had increased secretions and possible infiltrates on chest X-ray, as well as fever. For this reason, vancomycin and aztreonam were started (the patient had an allergy to cephalosporins) for the treatment of pneumonia. On day 4, the bronchoscopic culture showed Haemophilus influenzae. On day 7 of admission blood cultures grew corynebacterium afermentans. Ampicillin was added. On day 9, aztreonam was discontinued and cefepime started. On day 12, sputum culture showed Acinetobacter baumannii. Vancomycin, aztreonam, and ampicillin were discontinued, and she was treated for a total of seven days with ampicillin/sulbactam. Four weeks after admission, she had a positive sputum culture for Morganella morganii and Klebsiella pneumoniae for which she was treated with seven days of ceftriaxone. Urine and blood cultures remained negative.

From the cardiovascular standpoint, her blood pressure was labile and went from hypotensive to hypertensive requiring vasopressors and labetalol intermittently throughout hospitalization. From the pulmonary standpoint, she was ventilated on AC/VC+ with a tidal volume of 6ml/kilogram of ideal body weight, PEEP5, FI02 to maintain oxygen saturations >95%, and pC02 <32 mmHg. She developed pneumonia as stated above. Renal function remained normal throughout hospitalization.

Gastrointestinal and deep vein thrombosis prophylaxis was instituted with pantoprazole 40 mg oral daily and heparin 500 units subcutaneously every eight hours, respectively.

Several multidisciplinary meetings were attained with the patient’s family, MICU, obstetrics, neonatology, ethics, and social worker teams throughout hospitalization while somatic support was continued to maintain the viability of the pregnancy.

After 11 weeks since admission (week 33 of gestation), the fetal heart rate had significant decelerations with the baseline heart rate of 150-155 bpm with minimal variability, decreasing to 80 bpm lasting 5 mins. For this reason, the patient had an emergent low transverse cesarean section in the operating room. The infant was successfully delivered from a cephalic presentation weighting 2.142 kilograms, with APGAR (appearance, pulse, grimace, activity, and respiration) scores of 8 and 9. The mother was transported back to the MICU. The patient was subsequently disconnected from the ventilator. The infant did well after delivery and did not require resuscitation. She was brought to the neonatal intensive care unit for admission secondary to her gestational age. She was discharged home after five days.

## Discussion

The World Brain Death Project published in 2020 defines Brain Death/Death by Neurologic Criteria (BD/DNC) as the complete and permanent loss of brain function as defined by an unresponsive coma with loss of capacity for consciousness, brainstem reflexes, and the ability to breathe independently [3,4]. These guidelines recommend following BD/DNC protocols that have specific prerequisites, clinical testing, apnea testing, and possibly ancillary testing. Prerequisites include the presence of an irreversible, devastating brain injury leading to loss of all brain functions and there are no confounders (absence of medication or toxic effect, paralytics, severe electrolyte or acid-base or endocrine imbalance, facial or ocular trauma, hypoxia, and cervical spine instability). Clinical evaluation for the determination of BD/DNC comprises an assessment for coma and an evaluation for brainstem areflexia (fixed pupils, corneal, oculocephalic, and oculovestibular reflexes are absent, no facial movement to noxious stimulation, absent gag reflex, and no brain-mediated motor response to noxious stimulation of the limbs). Apnea testing assesses the function of the medulla by allowing carbon dioxide levels to rise and pH to fall sufficiently to maximally stimulate medullary respiratory centers. The absence of respiratory effort in response to hypercarbia and acidosis is consistent with brain death. The determination of BD/DNC can be made on clinical rounds. Ancillary testing should be conducted only when the clinical examination cannot be performed fully or safely. The recommended ancillary tests are digital subtraction angiography, radionuclear studies, and transcranial Doppler ultrasonography. It is suggested that brain CTA and MRA not be used. The time of death is typically the time at which the laboratory reports the values for arterial blood gas if the apnea test is performed or if ancillary testing is completed [3,5,6]. In our case, apnea testing was deferred due to the potential risk of acidosis to the fetus, thus, a nuclear medicine scan was pursued as ancillary testing, which met the criteria for brain BD/DNC.

The presence of pregnancy in a brain-dead woman poses a difficult clinical, ethical, and legal dilemma. On one hand, the woman is legally dead once declared BD/DNC. On the other hand, a decision can be made to continue somatic support to allow the maturity of the fetus until viability. From an ethical standpoint, the core principles of bioethics (beneficence, autonomy, nonmaleficence, and justice) would play a role for both the mother and the fetus. From the legal standpoint, a study by Lewis et al. showed that nearly 100% of hospital protocols do not address the identity of the decision maker for the fetus, leaving open the vast possibilities of the father, mother's next of kin, physicians, hospital administration, risk management, ethics committee, legal representative, or even the state [7]. In 2019, a study published by Cartolvni et al. proposed guidelines for the management of social and ethical aspects of brain death during pregnancy [7-9]. They proposed to adequately determine the mother's brain death state, assess the viability of the fetus, check with the father of the child, next of kin, family, and mother's previously expressed wishes, organize a combined meeting of the interdisciplinary team comprising physicians, caregiving team, hospital ethics committee, and members of the family to discuss fetal prognosis, mother's wishes, and emotional and financial factors, organize adequate support to the father, next of kin, or members of the family, seek to establish a decision within a clinical setting, avoiding the Court if possible, and only appeal to the Court in cases when a decision cannot be made between the members of the family or when it is perceived that their decision is not in the interests of the fetus and is contrary to good obstetric practice. Costs should not be underestimated; however, they should not be the primary concern and focus. Also, an international registry of brain-dead pregnant patients should be established [9].

In 2021, Gaia Dodaro et al. identified 35 cases in the literature of pregnancy while BD/DNC. The most common cause of BD/DNC was maternal intracranial hemorrhage, subarachnoid hemorrhage, and hematoma. The most common maternal complications were infections (pneumonia, urinary tract infection, and sepsis), diabetes insipidus, thermal variability, panhypopituitarism, and circulatory instability. Of the 35 cases, there were eight cases (23%) of intrauterine fetal demise and 27 neonates (77%) were born alive. Eight neonates (23%) were described as “healthy” at birth, 15 neonates (43%) had normal longer-term follow-up (>1 month to 8 years), two neonates (6%) had neurologic sequelae (from pre-term birth) and two neonates (6%) died [1]. Even though these numbers seem favorable, there is likely publication bias falsely elevating the survival rates, as cases with poor results are typically less likely to be published in favor of only reporting good outcomes.

The management of the pregnant BD/DNC patient can be compared with the management of the BD/DNC organ donor, although much more prolonged for pregnancy [10,11]. The most prevalent complications post BD/DNC include cardiac arrhythmias, diabetes insipidus, panhypopituitarism, disseminated intravascular coagulation, hypotension, neurogenic pulmonary edema, systolic myocardial dysfunction, and infections, among others [2].

From the endocrinologic standpoint, due to the resulting hormonal failure after brain death, hormone replacement therapy with thyroid hormone for hypothyroidism, steroids for adrenal insufficiency, and vasopressin for diabetes insipidus are widely used. Also, monitoring thyroid hormones and sodium, as well as urine output, is imperative [10]. From the cardiovascular standpoint, the combination of diabetes insipidus and the loss of sympathetic tone contribute to profound hypotension, which can be managed with fluid, hormone replacement, and vasopressors [10]. From the pulmonary standpoint, these patients are maintained on mechanical ventilation, ideally with a tidal volume of 6 ml/kg of ideal body weight to prevent acute respiratory distress syndrome [2,10]. In the pregnant patient, the target oxygen saturation is 95%. Due to the prolonged duration of ventilatory support, tracheostomy should be considered early to aid in tracheobronchial toileting, protect the airway from aspiration, and secure the airway, in spite of the possible complications of infection and bleeding [12,13]. Monitoring of infections and treatment with antibiotics is imperative to avoid the development of disseminated infections. Nutrition with tube feedings is important to maintain organs to allow the maturation and growth of the fetus [14].

## Conclusions

Once the diagnosis of BD/DNC is made for a pregnant woman, a difficult decision to continue with somatic support to maintain the fetus to viability has to be made. A multidisciplinary approach should be followed with the patient’s family and practitioners. If the decision is made to continue somatic support, the treatment of BD/DNC complications should be initiated, as well as continuous monitoring of the fetus.

BD/DNC is associated with several dysfunctions of the cardiac, respiratory, endocrinologic, and thermoregulatory systems. Even though literature in the past has shown reasonable outcomes of infants of BD/DNC mothers, there is likely publication bias falsely elevating the survival rates, as cases with poor outcomes are typically less likely to be published. We present a case of the successful delivery of an infant at 33 weeks of gestation after her mother was declared BD/DNC 11 weeks prior.
